# Machine learning classification of chronic traumatic brain injury using diffusion tensor imaging and NODDI: A replication and extension study

**DOI:** 10.1016/j.ynirp.2023.100157

**Published:** 2023-01-30

**Authors:** J. Michael Maurer, Keith A. Harenski, Subhadip Paul, Victor M. Vergara, David D. Stephenson, Aparna R. Gullapalli, Nathaniel E. Anderson, Gerard J.B. Clarke, Prashanth K. Nyalakanti, Carla L. Harenski, Jean Decety, Andrew R. Mayer, David B. Arciniegas, Vince D. Calhoun, Todd B. Parrish, Kent A. Kiehl

**Affiliations:** a The Mind Research Network, Albuquerque, NM, USA; b Department of Biomedical Science and Technology and Department of Sports Science, Ramakrishna Mission Vivekananda Educational and Research Institute (RKMVERI), West Bengal, India; c JIVAN – Centre for Research in Biological Sciences, Ramakrishna Mission Seva Pratishthan (RKMPSP), West Bengal, India; d Tri-Institutional Center for Translational Research in Neuroimaging and Data Science (TReNDS), Georgia State University, Georgia Institute of Technology, and Emory University, Atlanta, GA, USA; e Department of Psychology, Virginia Commonwealth University, Richmond, VA, USA; f Department of Psychology and Department of Psychiatry and Behavioral Neuroscience, University of Chicago, Chicago, IL, USA; g University of New Mexico Health Sciences Center, Albuquerque, NM, USA; h Marcus Institute for Brain Health, University of Colorado-Anschutz Medical Campus, Aurora, CO, USA; i Center for the Neurobiology of Language Recovery and Department of Radiology, Feinberg School of Medicine, Northwestern University, Evanston, IL, USA; j Departments of Psychology, Neuroscience, and Law, University of New Mexico, Albuquerque, NM, USA

**Keywords:** Traumatic brain injury, Machine learning, Pattern classifier, Fractional anisotropy, NODDI, Replication study

## Abstract

Individuals with acute and chronic traumatic brain injury (TBI) are associated with unique white matter (WM) structural abnormalities, including fractional anisotropy (FA) differences. Our research group previously used FA as a feature in a linear support vector machine (SVM) pattern classifier, observing high classification between individuals with and without acute TBI (i.e., an area under the curve [AUC] value of 75.50%). However, it is not known whether FA could similarly classify between individuals with and without history of chronic TBI. Here, we attempted to replicate our previous work with a new sample, investigating whether FA could similarly classify between incarcerated men with (*n* = 80) and without (*n* = 80) self-reported history of chronic TBI. Additionally, given limitations associated with FA, including underestimation of FA values in WM tracts containing crossing fibers, we extended upon our previous study by incorporating neurite orientation dispersion and density imaging (NODDI) metrics, including orientation dispersion (ODI) and isotropic volume (Viso). A linear SVM based classification approach, similar to our previous study, was incorporated here to classify between individuals with and without self-reported chronic TBI using FA and NODDI metrics as separate features. Overall classification rates were similar when incorporating FA and NODDI ODI metrics as features (AUC: 82.50%). Additionally, NODDI-based metrics provided the highest sensitivity (ODI: 85.00%) and specificity (Viso: 82.50%) rates. The current study serves as a replication and extension of our previous study, observing that multiple diffusion MRI metrics can reliably classify between individuals with and without self-reported history of chronic TBI.

## 1 Introduction

Traumatic brain injury (TBI) represents a significant global health concern, with worldwide estimates suggesting 69 million individuals sustain a TBI each year (Dewan et al., 2018). During the acute stages of TBI, damage occurs within myelinated white matter (WM) tracts, which connect spatially distinct brain regions and help contribute to efficient neurocognitive processing across multiple domains (Armstrong et al., 2016). Among the biomechanical forces occurring during acute TBI are rapid acceleration and deceleration of the brain within the skull, a consequence of which is diffuse axonal injury (DAI; also known as multifocal axonal injury [Meythaler et al., 2001]), or the shearing of axons within WM tracts (traumatic axonal injury [Povlishock and Katz, 2005]). Rather than simply reflecting recovery from an acute injury, TBI is increasingly being understood to reflect a chronic and potentially neurodegenerative disease (Green, 2016). For example, following traumatic axonal injury during acute TBI, specific proteins, including amyloid-β, start to build up within brain tissue, contributing to neurodegenerative processes (i.e., difficulty concentrating, memory difficulties) during later, chronic stages of TBI (Shahim and Zetterberg, 2022).

Individuals with acute and chronic TBI are characterized by different WM structural integrity abnormalities, which can be quantified using diffusion tensor imaging (DTI) metrics, including fractional anisotropy (FA). FA provides a measure of the degree of directionality of water diffusion (Mori and Zhang, 2006) and is a scalar value ranging from zero to one, with lower FA values representing reduced WM structural integrity. Studies with both youth and adults suggest that individuals with acute TBI are characterized by *increased* FA within WM tracts (Croall et al., 2014; Ling et al., 2012; Mayer et al., 2010; Roberts et al., 2014; Wilde et al., 2008), relating to increased cellular swelling (i.e., neuroinflammation) and reduced extracellular space (Chu et al., 2010; Pasternak et al., 2014). On the other hand, youth and adults with chronic TBI are routinely characterized by *reduced* FA (Croall et al., 2014; Kraus et al., 2007; Nakayama et al., 2006; Owens et al., 2018; Roberts et al., 2014; Xu et al., 2007), reflecting neurodegenerative processes, such as DAI and increased extracellular space (Bramlett and Dietrich, 2007; Gupta et al., 2005). In fact, diffusion MRI metrics of demyelination, including reduced FA, are a strong predictor of future neurodegeneration (Graham et al., 2020), suggesting that individuals with chronic TBI may be characterized by long-lasting, deleterious side effects following their initial injury.

Our research group previously investigated whether FA could be used to reliably classify between individuals with and without history of acute TBI (Vergara et al., 2017). In this previous study, participants with acute 10.13039/100013482TBI were scanned within three weeks of their initial injury, and FA values within WM voxels were used as a feature in a linear support vector machine (SVM) pattern classifier, resulting in high overall classification, measured via the area under the curve (AUC), of 75.50%. FA values within WM voxels also reliably classified between participants with acute TBI (sensitivity rate: 76.60%) and without acute TBI (specificity rate: 74.50%). However, it is not known whether similar WM structural integrity metrics could also reliably classify between individuals with and without history of chronic TBI, given different FA-related abnormalities associated with acute and chronic TBI.

The current study serves as a replication and extension of our previous study. Specifically, we attempted to replicate our previous study, investigating whether FA could reliably classify between individuals with and without history of self-reported chronic TBI in a new sample of incarcerated men using the linear SVM based classification approach included in our previous study. Additionally, we sought to extend upon our previous report, incorporating additional diffusion MRI metrics, using neurite orientation dispersion and density imaging (NODDI; Zhang et al., 2012). While FA is commonly used as a proxy measure for WM structural integrity, limitations are associated with this approach, including underestimation of FA values in WM tracts containing crossing fibers (Oouchi et al., 2007). Alternative diffusion MRI metrics, including NODDI, appear to be more reliable measures of structural integrity in WM tracts with more complex fiber organizations (Timmers et al., 2016) and are more sensitive in detecting TBI-related WM structural integrity deficits (Churchill et al., 2017; Palacios et al., 2020). We specifically investigated two NODDI metrics: the orientation dispersion index (ODI) of neurites (i.e., axons and dendrites), measuring the variability of neurite orientation, and the isotropic volume fraction (Viso), representing the free water content within the tissue (Zhang et al., 2012).

The purpose of this study was to evaluate whether our algorithmic approach would be applicable to a new sample of high-risk, incarcerated participants. Compared to individuals recruited from the general community, incarcerated individuals are characterized by a rate of TBI nearly five times higher (Schneider et al., 2021). Furthermore, incarcerated individuals with TBI history are more likely to recidivate once released compared to those without TBI history (Ray and Richardson, 2017). Here, we seek to use an objective, brain-based measure assessing WM structural integrity deficits in those characterized by chronic TBI. If our hypotheses are supported, this would help to identify participants with history of chronic 10.13039/100013482TBI that is associated with neuronal injury in this high-risk population, and to inform treatment intervention strategies. An important first step in this direction is identifying whether WM structural integrity measures, including FA, can reliably classify incarcerated individuals as having chronic TBI or not via machine learning. The current study serves as a replication and extension of our previous study (Vergara et al., 2017), investigating whether multiple diffusion MRI metrics (i.e., FA, NODDI ODI, and NODDI Viso) could reliably classify between incarcerated men with and without self-reported history of chronic TBI. We specifically hypothesized, consistent with our previous report, that FA would reliably classify between incarcerated men with and without history of self-reported history of chronic TBI. Additionally, we hypothesized that NODDI-based metrics would result in higher classification, especially sensitivity and specificity rates, compared to traditional WM structural integrity measures (i.e., FA), given previous research suggesting NODDI metrics may be more sensitive in identifying TBI-related abnormalities compared to FA (Churchill et al., 2017; Palacios et al., 2020).

## 2 Method

### 2.1 Participants

This study involved secondary analyses from data collected as part of a larger set of studies supported by multiple National Institute of Health (10.13039/100000002NIH) R01 awards, resulting in a large forensic database consisting of self-report and clinical assessments, neuropsychological tests, and structural and functional 10.13039/100011612MRI scans. Though these 10.13039/100000002NIH R01 awards explored different research topics (e.g., investigating the efficacy of different forms of substance use treatment, the structural and functional underpinnings of psychopathy, neural responses to drug-related stimuli, etc.), consistent assessments and 10.13039/100011612MRI scans (including DTI scans) were collected between studies. Exclusionary criteria for these previous studies included past or current history of CNS disease (e.g., strokes, tumors, repeated seizures, etc.), an estimated full-scale intelligence quotient (IQ) score less than 70, or less than a fourth-grade reading level.

For this specific study, 80 incarcerated men were randomly selected from this forensic database who previously completed a DTI scan and self-reported history of chronic TBI (i.e., reporting they experienced a TBI at least three months prior to DTI data collection [Wallace et al., 2018]). Additionally, 80 incarcerated men who had previously completed a DTI scan, but self-reported never experiencing a TBI, were randomly selected from this forensic database, to serve as an important comparison group. Though our previous report included men and women in analyses performed (Vergara et al., 2017), current analyses were restricted to only men, given significant gender differences related to TBI (Schneider et al., 2021). For example, while men are characterized by higher rates of TBI compared to women (Rutland-Brown et al., 2006), women experience more severe symptomatology following a TBI compared to men (Gupte et al., 2019). Therefore, analyses were restricted to only men, to ensure that gender did not introduce an additional confound in analyses performed (see also Anderson et al., 2019).

Participants (*n* = 160) ranged from 18 to 57 years of age at the time of DTI data collection (*M* = 36.91 years, *SD* = 10.19 years). On average, participants were incarcerated for a period of 4.49 years (*SD* = 5.61 years, range: 0.18 years–28.72 years) before DTI data collection. Participants with and without self-reported history of chronic TBI did not significantly differ with respect to age or incarceration length (see Table 1). Based on NIH race and ethnicity classifications, participants self-identified as American Indian or Alaskan Native (*n* = 9), Black or African American (*n* = 36), Native Hawaiian or other Pacific Islander (*n* = 2), White (*n* = 104), or more than one race (*n* = 3). Six participants chose not to self-disclose their race. Regarding ethnicity, participants self-identified as either Hispanic/Latino (*n* = 54) or non-Hispanic/Latino (*n* = 104); two participants chose not to self-disclose their ethnicity. Volunteer research participants provided written informed consent and were informed of their right to terminate participation at any point without consequence. Participants were compensated at a rate consistent with the hourly labor wage of the correctional facility at which they were housed. This research was approved by multiple IRBs, including the Ethical and Independent Review Services and the University of Wisconsin – Madison and the Office for Human Research Protections.

**Table 1:** Descriptive Statistics and Independent Samples t-tests between Participants with Self-Reported History of Chronic TBI (TBI+) and Participants without Self-Reported History of Chronic TBI (TBI-).

Variable	All Participants		TBI+		TBI-		Group Differences	
	M	SD	M	SD	M	SD	*t*	p
Age	36.91	10.19	36.94	10.27	36.88	10.17	0.04	.971
IQ	98.36	13.51	97.86	13.58	98.83	13.52	−0.44	.662
PCL-R Total	22.78	7.67	22.94	8.15	22.65	7.30	0.22	.827
Framewise Displacement	0.48	0.19	0.47	0.19	0.50	0.20	−0.82	.411
PTSD	1.13	0.46	1.17	0.50	1.09	0.40	1.12	.266
Number of Substance Dependencies	1.67	1.59	1.88	1.66	1.47	1.49	1.61	.109
Incarceration Length	4.49	5.61	4.77	5.72	4.19	5.55	0.49	.626

Race/ Ethnicity	N	% of Sample	N	% of Sample	N	% of Sample		

American Indian or Alaskan Native	9	5.63%	6	7.50%	3	3.75%		
Black or African American	36	22.50%	11	13.75%	25	31.25%		
Native Hawaiian or other Pacific Islander	2	1.25%	0	0.00%	2	2.5%		
White	104	65.00%	58	72.50%	46	57.50%		
More than One Race	3	1.88%	2	2.50%	1	1.25%		
Hispanic/Latino	54	33.75%	31	38.75%	23	28.75%		
Not Hispanic/Latino	104	65.00%	49	61.25%	55	68.75%		

*Note*. IQ refers to full-scale IQ estimates from the WAIS-III (Wechsler, 1997). PCL-R total refers to the total score derived from the Hare Psychopathy Checklist – Revised (PCL-R; Hare, 2003). Framewise displacement (FWD) refers to the average framewise displacement value across the entire DTI scan, to assess subject motion. PTSD refers to SCID-IV (First et al., 1997) PTSD scores (1 = not present, 2 = subthreshold, 3 = threshold). Number of substance dependencies refers to the total number of substance dependencies assessed via the SCID-IV (First et al., 1997). Incarceration length refers to the number of years from the participant's original sentence to their DTI scan. Additionally, we report number (and percentage) of participants who self-identified as each race and ethnicity category, for all participants, TBI + participants, and TBI- participants, respectively.

### 2.2 Assessments

#### 2.2.1 Traumatic brain injury (TBI)

TBI was assessed via self-report using a modified version of the Rivermead Post Concussion Symptoms Questionnaire (RPCSQ; King et al., 1995). Participants were asked whether they had ever sustained a TBI, the age and consequences of their injury, and the presence and duration of loss of consciousness associated with their injury. For participants who had self-reported experiencing a TBI, on average, they reported experiencing 1.86 TBIs (*SD* = 1.08, range: 1–5), with 20.30 years on average passing since their first self-reported TBI (*SD* = 11.71 years, range: 2.03–46.24 years). Furthermore, participants reported, on average, 15.38 years since their most recent TBI (*SD* = 11.86 years, range: 0.42–46.24 years).

#### 2.2.2 Additional assessments

In addition to TBI history, additional assessments were administered to ensure that groups did not significantly differ with respect to additional confounding variables, including IQ, psychopathic traits, and rates of post-traumatic stress disorder (PTSD) and substance use disorders (SUDs) (see Table 1). Full-scale IQ was estimated using the Vocabulary and Matrix Reasoning subtests from the Wechsler Adult Intelligence Scale – 3rd Edition (WAIS-III; Wechsler, 1997) (*M* = 98.36, *SD* = 13.51). While newer IQ assessments have since been developed (e.g., the Wechsler Abbreviated Scale of Intelligence – 2nd Edition [WASI-II; Wechsler, 2011), the WAIS-III is considered a reliable estimate of general intelligence (Ryan et al., 1999). Psychopathic traits, including a lack of empathy and impulsivity, were assessed using the Hare Psychopathy Checklist – Revised (PCL-R; Hare, 2003), a semi-structured interview designed to assess twenty different psychopathic traits across multiple domains of a person's life (*M* = 22.78, *SD* = 7.67). Individuals with chronic TBI and individuals scoring high on psychopathy are characterized by similar deficits, including empathic deficits (Wood and Williams, 2008) and increased impulsivity (Wood, 2017); as such, groups were matched on psychopathic traits. Past and present DSM-IV Axis I disorders were evaluated for all participants using the research version of the Structured Clinical Interview for DSM-IV Disorders (SCID-IV; First et al., 1997). Specifically, we investigated groups significantly differed with respect to rates of PTSD and SUDs, given the higher rate of these disorders among individuals who have sustained a TBI (Olsen and Corrigan, 2022; Vasterling and Dikmen, 2012). PTSD diagnoses were scored from one to three (one = not present, two = subthreshold, 3 = threshold) (*M* = 1.13, *SD* = 0.46). We investigated the number of substance dependencies each participant reported during the SCID-IV interview. Substance categories included: alcohol, cannabis, stimulants, sedatives/hypnotics/anxiolytics, cocaine, opioids, PCP, hallucinogens, solvents/inhalants, and other substances (*M* = 1.67, *SD* = 1.58).

### 2.3 Diffusion tensor imaging

Diffusion-weighted images were acquired at the correctional facilities where participants were housed using the Mind Research Network's 1.5T Siemens Avanto Mobile MRI scanner fitted with a 12-element head coil. A spin echo-planar imaging sequence was used with the following parameters: field of view = 256 × 256 mm, matrix size = 128 × 128, voxel size = 2.0 × 2.0 × 2.0 mm^3^, number of slices = 70 (no gap), TR/TE = 9200/84 ms, flip angle = 90°. Diffusion-weighted volumes were acquired using 30 non-collinear diffusion-sensitizing gradients (*b* = 800 s/mm^2^). Five interleaved non-diffusion weighted (*b* = 0 s/mm^2^) volumes were also collected to allow for motion and eddy current induced distortions and voxel-wise estimations of the elements of the diffusion tensor. Head motion was limited using padding and restraint during scans. All MRI scans were reviewed by an independent, licensed radiologist. Participants included in the current study were not characterized by any major brain abnormalities during radiological review. DTI motion parameters were quantified using framewise displacement (FWD); groups did not significantly differ with respect to FWD (see Table 1).

### 2.4 Processing of diffusion-weighted images

Diffusion-weighted data were first converted from DICOM to NIFTI using the *dcm2niix* program (https://www.nitrc.org/projects/mricron/). Tables of *b* values and *b* vectors from the data were extracted for further processing. Eddy current and motion correction was performed using the *eddy_cuda8* function (Andersson and Sotiropoulos, 2016), available in the Oxford Centre for FMRIB Software Library (FSL 6.0.3: https://www.fmrib.ox.ac.uk/fsl/). The rotational parameters of the affine transformation were used to rotate the gradient vectors after artifact correction (Leemans and Jones, 2009). Brain extraction using the *b* = 0 vol, estimation of the diffusion tensor elements, and voxel-wise FA mapping were performed using the brain extraction tool and FDT utilities within FSL, respectively (Jenkinson et al., 2012). FA maps were non-linearly warped to the FMRIB58 1.0 × 1.0 × 1.0 mm^3^ FA template using the *fsl_reg* function. Diffusion data quality control was assessed using the *eddy_quad* function for individual subjects and the *eddy_squad* function for the full sample and each subsample (i.e., TBI+ and TBI-) (Bastiani et al., 2019). Warped FA images were averaged and a WM mask was created by thresholding the averaged FA image at 0.20. After regressing out participant's age from FA estimates of WM voxels, the voxel-based residual FA was used in subsequent classification analyses. Age was regressed out of FA estimates of WM voxels to be consistent with our previous report (Vergara et al., 2017), which regressed out age and gender in analyses performed. As our sample was restricted to only men, we regressed out only age from diffusion MRI metrics, rather than other variables collected (e.g., psychopathy, IQ, substance use, etc.) to be consistent with our previous study.

The NODDI technique (Zhang et al., 2012) was also used to investigate a three-component geometric model of diffusion. This advanced approach models intracellular and extracellular water diffusion separately, allowing for the estimation of fractional water contributions of different tissue types within each voxel. NODDI data was generated using the Microstructure Diffusion Toolbox (MDT; Harms and Roebroeck, 2018). The ODI and Viso compartment data were warped to the same standard FMRIB FA template as the voxel-based FA using FSL's *applywarp* routine, and then masked with a WM segmentation map, to only include WM voxels, with age regressed out of the remaining voxels, and residual maps used for subsequent classification analyses. Previously published studies suggest that NODDI metrics, including ODI and Viso, are reliable using single-shell, low b-value data (Cottaar et al., 2019; Lucignani et al., 2021; Timmers et al., 2016; Zhang et al., 2012) and through the use of MRI scanners with low field strength (Chung et al., 2016).

### 2.5 Support vector machine based classification

Machine learning decisions were made consistent with our previously published study (Vergara et al., 2017). For example, a linear SVM pattern classifier approach with nested leave-one-out cross-validation (LOOCV) was used for the classification of TBI+ and TBI- groups, with FA, NODDI ODI, and NODDI Viso used as features in separate analyses. Important to note, we did not apply this previously trained linear SVM pattern classifier to the current sample, given differences related to sample composition and field strength. Instead, we trained the linear SVM classifier using the same algorithmic approach in a new, independent sample of incarcerated men with and without self-reported history of chronic TBI. The least squares (LS) method and sequential minimal optimization (SMO) methods were used to solve the linear SVM. The SVM model was tuned by evaluating across six different soft-margin parameter values (0.05, 0.1, 0.5, 1.0, 5.0, and 10.0). Consistent with our previous study (Vergara et al., 2017), *t*-values related to significant differences between the TBI+ and TBI- groups were used to evaluate the SVM models with reduced features. The maximum AUC measure was used for model parameter selection for each of the cross-validation folds. Using the criterion of maximum frequency of selection, the LS method was used to solve the linear SVM, with a soft-margin parameter of C = 0.05, and *t*-threshold of 2.0 for both voxel-based FA and NODDI (ODI and Viso) data.

## 3 Results

### 3.1 Classification results

*Voxel-Based FA*: High classification rates were observed when incorporating traditional DTI metrics (i.e., voxel-based FA) as a feature in classification analyses (see Table 2). Specifically, FA resulted in a high overall classification rate across all participants (i.e., an AUC value of 82.50%), the TBI + group (sensitivity: 83.75%), and TBI- group (specificity: 81.25%).

**Table 2:** Classification rates of different diffusion MRI metrics.

Feature	Area Under the Curve (AUC)	Sensitivity	Specificity
FA	82.50%	83.75%	81.25%
NODDI ODI	82.50%	85.00%	80.00%
NODDI Viso	77.50%	72.50%	82.50%

*Note*. FA refers to DTI-based fractional anisotropy; NODDI ODI refers to orientation dispersion values obtained from NODDI; NODDI Viso refers to free water metrics obtained from NODDI. The area under the curve was used to assess overall classification rates. Sensitivity refers to the accuracy of the model in classifying individuals with self-reported history of chronic TBI (TBI+) and specificity refers to the accuracy of the model in classifying individuals without self-reported history of chronic TBI (TBI-).

When using FA as a feature in the linear SVM pattern classifier, 67 participants were correctly classified as TBI+ and 65 participants were correctly classified as TBI-, whereas 13 participants were incorrectly classified as TBI+ and 15 participants were incorrectly classified as TBI- (see Table 3). When comparing TBI+ participants who were correctly and incorrectly classified, groups significantly differed with respect to rates of PTSD, with those misclassified being more likely to score subthreshold or above threshold on PTSD, compared to those who were correctly classified. Groups did not significantly differ with respect to other variables investigated (see Table 4). Additionally, when comparing TBI- participants who were correctly and incorrectly classified, groups did not significantly differ with respect to any of the variables investigated (see Table 5).

**Table 3:** Number of participants correctly classified as TBI+ and TBI- with different diffusion MRI metrics.

Feature	Number Correctly Classified as TBI+	Number Correctly Classified as TBI-	Number Incorrectly Classified as TBI+	Number Incorrectly Classified as TBI-
FA	67	65	13	15
NODDI ODI	68	64	12	16
NODDI Viso	58	66	22	14

*Note*. FA refers to DTI-based fractional anisotropy; NODDI ODI refers to orientation dispersion values obtained from NODDI; NODDI Viso refers to free water metrics obtained from NODDI.

**Table 4:** Comparing TBI+ Participants who were Correctly and Incorrectly Classified.

Variable	Incorrectly Classified		Correctly Classified		Group Differences	
	M	SD	M	SD	*t*	p
Age	36.88	4.33	36.95	11.08	−0.02	.983
IQ	98.75	15.66	97.69	13.27	0.25	.807
PCL-R Total	22.29	8.87	23.07	8.07	−0.29	.775
Framewise Displacement	0.45	0.14	0.48	0.19	−0.49	.629
PTSD	1.46	0.78	1.11	0.41	2.34	.022
Number of Substance Dependencies	2.15	1.72	1.82	1.65	0.65	.517
Incarceration Length	6.55	3.80	4.36	6.05	1.04	.304
Total Number of TBIs	1.92	1.26	1.85	1.05	0.22	.826
Time Since First TBI	19.06	10.02	20.53	12.05	−0.40	.692
Time Since Last TBI	15.90	8.75	15.28	12.41	0.16	.871

Race/ Ethnicity	N	% of Sample	N	% of Sample		
American Indian or Alaskan Native	2	15.38%	4	5.97%		
Black or African American	2	15.38%	9	13.43%		
Native Hawaiian or other Pacific Islander	0	0.00%	0	0.00%		
White	7	53.85%	51	76.12%		
More than One Race	1	7.69%	1	1.49%		
Hispanic/Latino	6	46.15%	25	37.31%		
Not Hispanic/Latino	7	53.85%	42	62.69%		

*Note*. *Note*. IQ refers to full-scale IQ estimates from the WAIS-III (Wechsler, 1997). PCL-R total refers to the total score derived from the Hare Psychopathy Checklist – Revised (PCL-R; Hare, 2003). Framewise displacement (FWD) refers to the average framewise displacement value across the entire DTI scan, to assess subject motion. PTSD refers to SCID-IV (First et al., 1997) PTSD scores (1 = not present, 2 = subthreshold, 3 = threshold). Number of substance dependencies refers to the total number of substance dependencies assessed via the SCID-IV (First et al., 1997). Incarceration length refers to the number of years from the participant's original sentence to their DTI scan. Time Since 1^st^ TBI refers to the amount of time between the participant's first self-reported TBI and their DTI scan. Time Since Last TBI refers to the amount of time between the participant's most recent self-reported TBI and their DTI scan. Additionally, we report number (and percentage) of participants who self-identified as each race and ethnicity category, for participants who were incorrectly and correctly classified as TBI+, respectively.

**Table 5:** Comparing TBI- Participants who were Correctly and Incorrectly Classified.

	Incorrectly Classified		Correctly Classified		Group Differences	
Variable	M	SD	M	SD	*t*	p
Age	39.07	9.28	36.37	10.36	0.93	.357
IQ	99.67	8.27	98.63	14.55	0.27	.792
PCL-R Total	21.47	7.31	22.92	7.33	−0.67	.507
Framewise Displacement	0.49	0.21	0.50	0.19	−0.11	.910
PTSD	1.00	0.00	1.11	0.45	−0.97	.334
Number of Substance Dependencies	0.93	1.33	1.60	1.51	−1.56	.123
Incarceration Length	4.82	6.52	4.08	5.44	0.32	.751

Race/ Ethnicity	N	% of Sample	N	% of Sample		

American Indian or Alaskan Native	1	6.67%	2	3.08%		
Black or African American	6	40.00%	19	29.23%		
Native Hawaiian or other Pacific Islander	1	6.67%	1	1.54%		
White	7	46.67%	39	60%		
More than One Race	0	0.00%	1	1.54%		
Hispanic/Latino	1	6.67%	22	33.85%		
Not Hispanic/Latino	14	93.33%	41	63.08%		

*Note*. *Note*. IQ refers to full-scale IQ estimates from the WAIS-III (Wechsler, 1997). PCL-R total refers to the total score derived from the Hare Psychopathy Checklist – Revised (PCL-R; Hare, 2003). Framewise displacement (FWD) refers to the average framewise displacement value across the entire DTI scan, to assess subject motion. PTSD refers to SCID-IV (First et al., 1997) PTSD scores (1 = not present, 2 = subthreshold, 3 = threshold). Number of substance dependencies refers to the total number of substance dependencies assessed via the SCID-IV (First et al., 1997). Incarceration length refers to the number of years from the participant's original sentence to their DTI scan. Time since 1^st^ TBI refers to the amount of time between the participant's first self-reported TBI and their DTI scan. Time since Last TBI refers to the amount of time between the participant's most recent self-reported TBI and their DTI scan. Additionally, we report number (and percentage) of participants who self-identified as each race and ethnicity category, for participants who were incorrectly and correctly classified as TBI-, respectively.

*NODDI ODI*: NODDI ODI features also resulted in high classification rates (see Table 2). NODDI ODI was associated with a similar overall classification rate as FA (i.e., AUC value of 82.50%), but higher sensitivity (85.00%) and lower specificity rates (80.00%). When using NODDI, 68 participants were correctly classified as TBI+ and 64 participants were correctly classified as TBI-, whereas 12 participants were incorrectly classified as TBI+ and 16 participants were incorrectly classified as TBI- (see Table 3).

*NODDI Viso*: Compared to FA and NODDI ODI, the use of NODDI Viso features resulted in lower overall classification rate (AUC value of 77.50%) and sensitivity rates (72.50%) (see Table 2). However, using NODDI Viso features, higher specificity rates were observed (82.50%) compared to other diffusion MRI metrics. When using NODDI Viso, 58 participants were correctly classified as TBI+ and 66 participants were correctly classified as TBI-, whereas 22 participants were incorrectly classified as TBI+ and 14 participants were incorrectly classified as TBI- (see Table 3).

Across the three diffusion MRI metrics, there was substantial consistency between the participants who were misclassified. For example, of the 13 participants with a TBI who were classified as TBI- using FA, 7 were also misclassified using NODDI ODI (54%) and 10 were misclassified using NODDI Viso (77%). Additionally, for the 15 participants without a TBI who were incorrectly classified as having a TBI, 11 were misclassified using NODDI ODI (73%) and 12 were misclassified using NODDI Viso (80%). Therefore, rather than combining FA and NODDI metrics as features into the same linear SVM pattern classifier, it appears as if some participants are similarly misclassified, regardless of the specific features or feature set used.

## 4 Discussion

Our research group previously investigated whether DTI metrics, specifically FA, could reliably classify between participants with and without acute TBI using a linear SVM pattern classifier (Vergara et al., 2017). We previously observed high classification rates when incorporating FA as a feature in a linear SVM pattern classifier, including overall classification rate (i.e., AUC value of 75.50%) and sensitivity (76.60%) and specificity (74.50%) rates. However, given different FA-related abnormalities between individuals with acute and chronic TBI among youth and adults (Croall et al., 2014; Ling et al., 2012; Kraus et al., 2007; Mayer et al., 2010; Nakayama et al., 2006; Owens et al., 2018; Roberts et al., 2014; Wilde et al., 2019; Xu et al., 2007), it remains to be seen whether diffusion MRI metrics could help reliably classify between individuals with and without chronic TBI. Here, we attempted to replicate and extend upon our previous study, specifically investigating whether FA and NODDI metrics (e.g., ODI and Viso) could reliably classify between individuals with and without history of self-reported chronic TBI in a new sample of incarcerated men. We observed that the highest overall classification rates were obtained using multiple diffusion MRI metrics, specifically FA and NODDI ODI, with AUC values of 82.50%. Additionally, NODDI metrics resulted in the highest sensitivity rate (predicting TBI + participants with 85.00% accuracy using NODDI ODI) and specificity rate (predicting TBI- participants with 82.50% accuracy using NODDI Viso). Therefore, the current study serves as a replication and extension of our previous study (Vergara et al., 2017), where multiple diffusion MRI metrics were able to accurately classify between incarcerated individuals with and without self-reported history of chronic TBI.

Current approaches to identify TBI history in incarcerated individuals often rely upon the use of self-report assessments. While incarcerated individuals have been shown to report generally accurate histories of TBI compared to hospital medical records (Schofield et al., 2011), incarcerated individuals are characterized by a higher overall number of TBIs compared to individuals recruited from the general community (Schneider et al., 2021). This, combined with previous evidence suggesting poor recall accuracy among individuals with multiple TBIs (McKinlay et al., 2016), suggests that incarcerated individuals may underreport history of TBI, especially TBIs occurring early in life. Therefore, by using objective, brain-based measures of WM structural integrity metrics, we may be able to classify incarcerated individuals more reliably as having previously sustained a TBI or not. For example, as reported in Table 3, when incorporating FA as a feature in the linear SVM pattern classifier, 15 individuals included in the TBI- group were misclassified as having a TBI. Similar results were observed when using NODDI metrics, with 16 and 14 participants misclassified using NODDI ODI and Viso, respectively. By correctly identifying incarcerated individuals with TBI history, even unknown, specialized treatment intervention approaches can be developed specifically for this population, including mindfulness-based approaches. Previous evidence suggests that mindfulness has been associated with improvements in individuals with TBI (Acabchuk et al., 2021) and reducing antisocial outcomes, including recidivism (Alexander et al., 2003).

Extending beyond our previous study (Vergara et al., 2017), we also observed that additional diffusion MRI metrics (i.e., NODDI ODI and Viso) were associated with the highest sensitivity (85.00%) and specificity (82.50%) rates. Lower sensitivity (83.75%) and specificity (81.25%) rates were observed when incorporating traditional DTI FA metrics. Such results are consistent with previously published studies, suggesting that NODDI-based metrics may be more sensitive in identifying TBI-related WM structural integrity deficits compared to traditional DTI measures (Churchill et al., 2017; Palacios et al., 2020). Important to note, as diffusion MRI data was collected using a 12-element head coil in the current study, other NODDI metrics, including intracellular (Vic) compartment and neurite density index (NDI) models, were not investigated, given previous evidence suggesting NODDI metrics are poorly estimated when extracted from single shell data (Zhang et al., 2012). Therefore, there exists the possibility with alternative head coils (e.g., 32-channel head coils), classification accuracy would improve using additional NODDI-based metrics.

### 4.1 Limitations

Additional limitations associated with the current study should be noted. First, we estimated NODDI Viso using single-shell, low *b*-value diffusion data. While Viso single-shell maps can be noisy, they are not significantly biased for low *b*-values (Zhang et al., 2012). The single shell data used in the current study did not have high weighted *b*-values (*b* = 800); as such, we opted to investigate whether NODDI Viso could reliably classify between groups. It remains to be seen whether NODDI Viso would also reliably classify between participants with and without chronic TBI when incorporating high *b*-value diffusion data. Second, we did not investigate whether other DTI metrics, including radial diffusivity (RD) and axial diffusivity (AD) were associated with higher classification rates compared to FA. RD and AD have been shown to relate to TBI (Khong et al., 2016; Mahan et al., 2021); however, FA was included as the primary DTI metric in the current study, to see whether similar classification rates were observed as our previous study classifying individuals with and without acute TBI recruited from the general community (Vergara et al., 2017). Additionally, we only investigated whether diffusion MRI metrics could reliably classify between participants with and without self-reported history of chronic TBI in incarcerated men, not incarcerated women. Future work is planned examining whether our current results generalize to incarcerated women. Finally, individuals without self-reported history of TBI served as our comparison group. While some studies have compared individuals with history of TBI to uninjured participants, such group differences may be moderated by other differences inherent to individuals with TBI, including neurobehavioral characteristics (e.g., impulsivity and rates of PTSD). More recent studies have compared individuals with TBI to individuals with previous orthopedic injury, observing non-significant group differences for FA (Wilde et al., 2019) and NODDI metrics (Shukla et al., 2021). While we ensured that TBI+ and TBI- groups did not significantly differ with respect to multiple variables known to influence WM structural integrity, including psychopathic traits, rates of PTSD and SUDs, and IQ, there exists the possibility that an unmeasured variable may moderate the current results. This issue is germane to any between-subjects study, and future research should consider examining other potential moderating variables (e.g., childhood trauma, fetal alcohol exposure, etc.).

### 4.2 Conclusions

The current study serves as a replication and extension of our previous study (Vergara et al., 2017), observing that multiple diffusion MRI metrics, including traditional DTI metrics (i.e., FA) and NODDI-based measures (i.e., NODDI ODI and Viso), could reliably classify between incarcerated individuals with and without self-reported history of chronic TBI. Similar overall classification rates were observed between DTI FA and NODDI ODI metrics (i.e., AUC values of 82.50%). Furthermore, the highest sensitivity and specificity rates were observed using NODDI-based metrics as features. Therefore, across multiple studies performed by our research group, diffusion MRI metrics have shown to reliably classify individuals as TBI + or TBI- across different samples (e.g., community and incarcerated participants), scanner field strength (i.e., 3.0T and 1.5T), and timing since original TBI (i.e., acute and chronic TBI).

## Declaration of competing interest

The authors declare that they have no known competing financial interests or personal relationships that could have appeared to influence the work reported in this paper.

## Data Availability

Data will be made available on request.
